# Comparison of Poly-A^+^ Selection and rRNA Depletion in Detection of lncRNA in Two Equine Tissues Using RNA-seq

**DOI:** 10.3390/ncrna6030032

**Published:** 2020-08-21

**Authors:** Anna R. Dahlgren, Erica Y. Scott, Tamer Mansour, Erin N. Hales, Pablo J. Ross, Theodore S. Kalbfleisch, James N. MacLeod, Jessica L. Petersen, Rebecca R. Bellone, Carrie J. Finno

**Affiliations:** 1 Department of Population Health and Reproduction, School of Veterinary Medicine, University of California Davis, Davis, CA 95616, USA; adahlgren@ucdavis.edu (A.R.D.); drtamermansour@gmail.com (T.M.); enburns@ucdavis.edu (E.N.H.); rbellone@ucdavis.edu (R.R.B.); 2 Department of Animal Science, College of Agricultural and Environmental Sciences, University of California Davis, Davis, CA 95616, USA; ericay.scott@utoronto.ca (E.Y.S.); pross@ucdavis.edu (P.J.R.); 3 Gluck Equine Research Center, Department of Veterinary Science, University of Kentucky, Lexington, KY 40546, USA; ted.kalbfleisch@uky.edu (T.S.K.); jnmacleod@uky.edu (J.N.M.); 4 Department of Animal Science, University of Nebraska Lincoln, Lincoln, NE 68583, USA; jessica.petersen@unl.edu; 5 Veterinary Genetics Laboratory, School of Veterinary Medicine, University of California Davis, Davis, CA 95616, USA

**Keywords:** annotation, transcriptome, regulatory, horse

## Abstract

Long non-coding RNAs (lncRNAs) are untranslated regulatory transcripts longer than 200 nucleotides that can play a role in transcriptional, post-translational, and epigenetic regulation. Traditionally, RNA-sequencing (RNA-seq) libraries have been created by isolating transcriptomic RNA via poly-A^+^ selection. In the past 10 years, methods to perform ribosomal RNA (rRNA) depletion of total RNA have been developed as an alternative, aiming for better coverage of whole transcriptomic RNA, both polyadenylated and non-polyadenylated transcripts. The purpose of this study was to determine which library preparation method is optimal for lncRNA investigations in the horse. Using liver and cerebral parietal lobe tissues from two healthy Thoroughbred mares, RNA-seq libraries were prepared using standard poly-A^+^ selection and rRNA-depletion methods. Averaging the two biologic replicates, poly-A^+^ selection yielded 327 and 773 more unique lncRNA transcripts for liver and parietal lobe, respectively. More lncRNA were found to be unique to poly-A^+^ selected libraries, and rRNA-depletion identified small nucleolar RNA (snoRNA) to have a higher relative expression than in the poly-A^+^ selected libraries. Overall, poly-A^+^ selection provides a more thorough identification of total lncRNA in equine tissues while rRNA-depletion may allow for easier detection of snoRNAs.

## 1. Introduction

Long non-coding RNAs (lncRNAs) are untranslated transcripts longer than 200 nucleotides (nt). They have been shown to have a wide range of functions in the regulation of transcription, translation, epigenetics, differentiation, and the cell cycle [1,2,3,4,5,6,7]. In recent years, lncRNAs have been increasingly shown to play important roles in diseases, particularly cancer [8,9,10] and neurodegeneration [11,12]. However, many functional roles of lncRNAs in cell biology, development, and disease pathogenesis remain unknown, especially in the horse. As sequence conservation of lncRNA among species is low [13], lncRNAs identified in the human and mouse often are not expected to have a similar genomic sequence in horses.

Previously, an equine lncRNA pipeline and database (https://github.com/eyscott/lncRNA) was developed using RNA sequencing (RNA-seq) data from various laboratories and across disease phenotypes [13]. While filling a necessary gap in knowledge in equine genetics, the database had some limitations. One of the largest limitations was that many of the horses had one of several diseases. This prevents the public database from being used as a baseline from which to identify aberrant lncRNA expression or splicing in disease-affected horses. Additionally, some RNA samples were prepared using ribosomal RNA (rRNA)-depletion whereas other samples were prepared with poly-A^+^ selection. However, both methods were not used on any single tissue, so there was no way to assess and quantify different transcription profiles as a function of the library preparation methods used. Previous human studies have demonstrated that the transcripts that are sequenced may differ in quantity and identity between the two methods, with poly-A^+^ selection limited to transcripts with a polyadenylated tail and rRNA-depleted libraries having the additional challenge of often including intronic and intergenic regions [14]. In the horse, biologic validation of putative lncRNAs is lacking; therefore, it is difficult to distinguish between novel lncRNA and true intronic and intergenic reads.

The objective of this study was to determine which RNA-seq library preparation method would most reliably capture lncRNA in the equine genome. Long ncRNA was the focus of this study since substantial work is already in progress to annotate equine protein-coding genes, lncRNA are more likely than protein-coding genes to differ from other species [13,15,16,17], and there are potentially fewer lncRNA with poly-A tails. Using liver and cerebral parietal lobe tissues collected from two healthy Thoroughbred mares as part of the Functional Annotation of Animal Genomes (FAANG) initiative, direct comparisons between library preparations was performed. These two tissues were chosen to be representative of homogenous and heterogeneous tissue, respectively. As non-polyadenylated lncRNA have been identified in other species [18,19], our hypothesis was that rRNA-depleted libraries would be preferable for annotating lncRNA as this method is not dependent on the transcripts being polyadenylated. Determining the RNA-seq library preparation method best for identifying lncRNA is an essential step toward annotation of the horse genome to identify genetic regions and variants associated with diseases.

## 2. Results

Liver and parietal lobe of the cerebrum were collected from two healthy Thoroughbred mares (adult horse 1: AH1 and adult horse 2: AH2) [20]. RNA was isolated and prepared for sequencing with poly-A^+^ selection and rRNA-depletion. Four filters were applied to the resulting RNA-seq datasets as previously described [13] to isolate lncRNA transcripts. First, single exon transcripts with low transcripts per million (TPMs) were filtered out as done previously [13] to remove likely uninformative reads and polymerase mistakes. Then, known protein-coding transcripts were filtered out. Next, the remaining transcripts were filtered based on the definition of lncRNA (>200 nt) and by TPM. To ensure no protein-coding transcripts remain, protein-coding transcripts were computationally predicted and removed. Lastly, previous work has shown that this pipeline removes some true lncRNA, so a rescue step is required [13]. This was done by comparing the removed transcripts to known human lncRNA.

**Figure 1:**
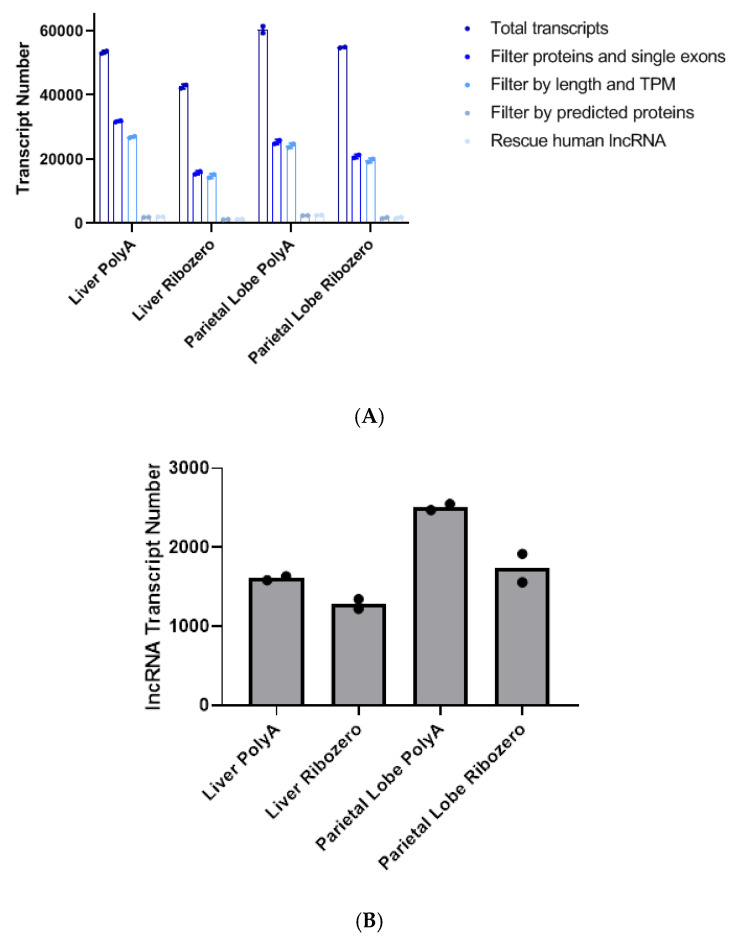
(**A**) Bar graph showing the average number of total transcripts between the two horses after each filtering step. TPM = transcripts per million. (**B**) Number of unique long non-coding RNAs (lncRNA) in each tissue library preparation combination (same as the last bar in A). Each data point represents one horse. PolyA indicates poly-A^+^ selection.

Filtering out known protein-coding and single exon transcripts expressed at low levels resulted in the greatest removal of transcripts for all the samples (Figure 1A). Libraries prepared with rRNA-depletion had more protein-coding transcripts removed across tissues and biologic replicates (Figure S1), and poly-A^+^ selection yielded more unique lncRNA (Figure 1B). Additionally, as expected, the more complex parietal lobe samples had more unique lncRNA transcripts than the liver samples, which have a more homogenous cellular composition (Figure 1B).

**Figure 2:**
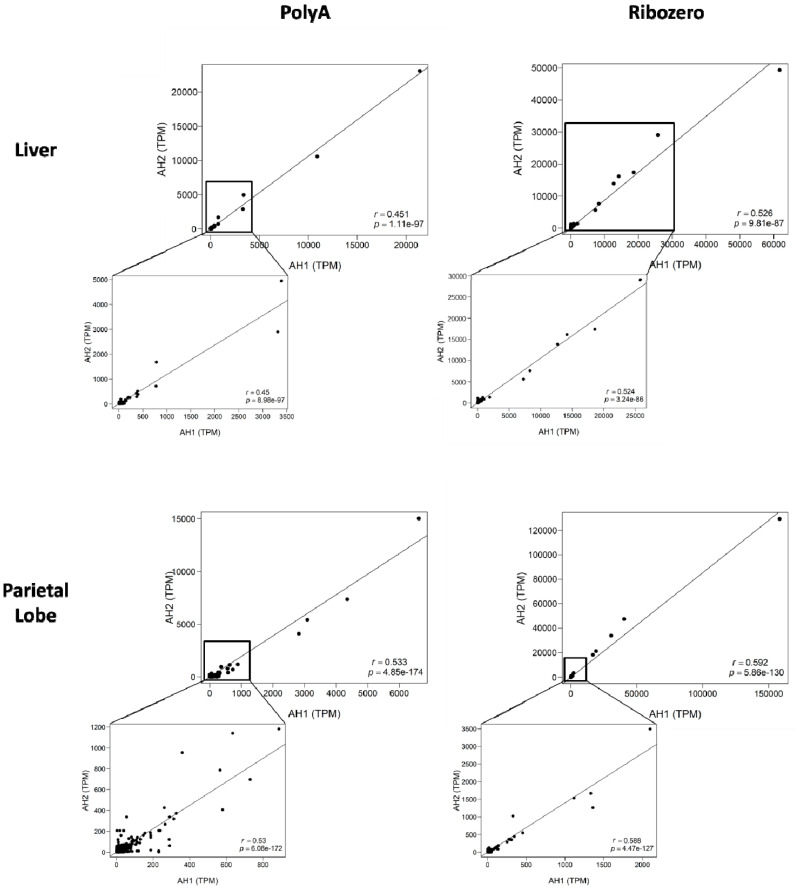
Correlation plots for lncRNA expression between biologic replicates. The poly-A^+^ selected libraries are on the left, the rRNA depleted libraries on the right. The top two graphs are from the liver while the bottom two are from the parietal lobe of the cerebrum. The x-axis indicates expression of the individual lncRNA in adult horse 1 (AH1). The y-axis shows expression of the same lncRNAs in adult horse 2 (AH2). Spearman correlation (r) and *p*-value in bottom right corner.

To investigate if the lncRNA expression was similar between the two biologic replicates, we plotted the TPM values for each horse against each other for each tissue and library preparation method in a correlation plot. Analogous unannotated transcripts between biologic replicates were identified via bedtools intersect (Table S1). Correlation was significant across both tissue types (Figure 2). In each dataset, there were unique transcripts that were outliers (Table S2) with high TPM values. However, even when the outliers were removed, correlations of biologic replicates between library preparations remained significant (Spearman r_(liver_polyA)_ = 0.45, *p* = 8.98 × 10^−97^; Spearman r_(liver_ribo)_ = 0.524, *p* = 3.24 × 10^−86^; Spearman r_(parietal_polyA)_ = 0.53, *p* = 6.08 × 10^−172^; Spearman r_(parietal_ribo)_ = 0.588, *p* = 4.47 × 10^−127^). Taken together, these findings demonstrate strong correlation of biologic replicates within library preparations and tissue types.

Differentially expressed (DE) lncRNAs between liver and parietal lobe samples for each library preparation were determined (Table S3). While most of the DE lncRNA were unannotated, *H19* was identified by both poly-A^+^ selection and rRNA-depletion as being expressed higher in the liver than the parietal lobe, similar to findings in humans [21]. In poly-A^+^ selected libraries, lncRNAs that appear similar to *LINC00643*, *LINC02586*, and *LOC100128494* in humans have similar expression patterns in liver and parietal lobe [21]. For example, *LINC00643* is highly expressed in the brain in humans and only minimally in the liver [21], which parallels what we see in our RNA-seq data. In rRNA-depleted libraries, lncRNAs that have similarities in sequence or genomic position to *MIR124-2HG*, *LINC00643*, *RP4-785G19.5*, and *LINC01351* in humans have parallel expression patterns in liver and parietal lobe in the horse [21]. *H19, LINC02586, MIR124-2HG,* and *LINC01351* expression in the parietal lobe and liver was confirmed with quantitative reverse transcription PCR (qRT-PCR) in the same horses (Figure S2). This suggests that both library preparations are accurately demonstrating relative expression between tissue types.

Comparing the DE lncRNAs that are annotated by National Center for Biotechnology Information (NCBI) showed that the top two lncRNA are the same for poly-A^+^ selection and rRNA-depletion. One in an unknown lncRNA (rna69770; Table S3) and the other is *H19* (rna41570; Table S3). Additionally, within the top 10 DE lncRNA, there are two other lncRNA that show up in both library preparations. There is another unknown lncRNA (rna12060; Table S3) and the other is similar to human *LINC00643* (rna64504; Table S3). So, only four of the top 10 annotated DE lncRNA are the same between library preparations, indicating that there is a substantial difference in the quantity of lncRNA that is detected by each library preparation.

**Figure 3:**
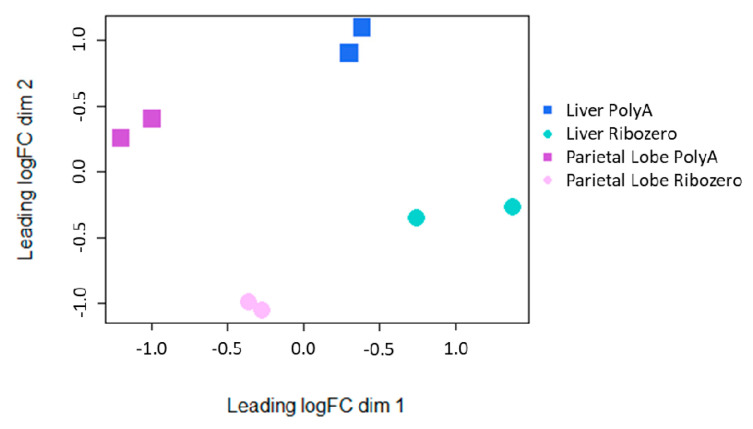
Multidimensional scaling plot of the lncRNA expression from each tissue/library preparation for each horse.

To further address the impact that library preparation plays in defining the lncRNA transcriptome, a multi-dimensional scaling (MDS) plot was evaluated. While tissue type caused the largest difference between samples (dimension 1; x-axis), library preparation caused the second largest difference (dimension 2; y-axis; Figure 3). Principal component analysis (PCA) also showed an even greater difference between tissues when rRNA-depletion was used (PC1 = 70.9%, PC2 = 12%; Figure S3).

We also investigated how many lncRNAs only appear in one library preparation. Within poly-A^+^ selected libraries for the liver and parietal lobe, 1276 and 2602 unique lncRNA were identified, respectively. Fewer lncRNA were unique to the rRNA-depleted libraries, with 977 and 1467 lncRNAs identified for the liver and parietal lobe, respectively. This suggests poly-A^+^ selection captures more lncRNA than rRNA-depletion.

**Figure 4:**
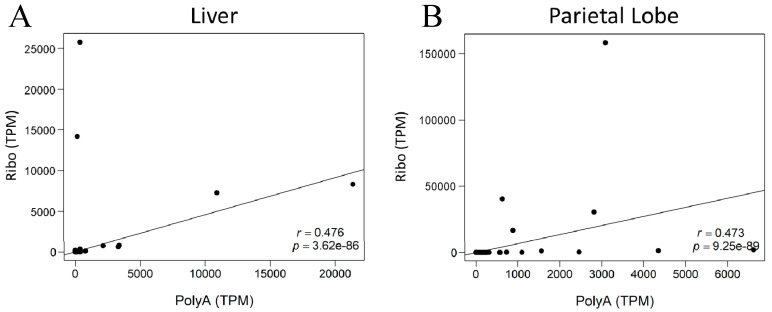
Correlation plots for lncRNA expression in AH1 with annotated transcripts. The x-axis indicates expression of the individual lncRNA in the poly-A^+^ selected library. The y-axis shows expression of the same lncRNAs in the rRNA-depleted library. (**A**) Liver (**B**) Parietal lobe of the cerebrum. Spearman correlation (r) and *p*-value in bottom right corner.

To continue investigating the differences between library preparations, correlation plots were constructed between library preparation methods for each horse and each tissue (Figure 4 and Figure S4). There was only a moderate degree of correlation between library preparations (Spearman r_(liver,subset)_ = 0.476, *p* = 3.62 × 10^−86^; Spearmen r_(parietal,subset)_ = 0.473, *p* = 9.25 × 10^−89^).

**Figure 5:**
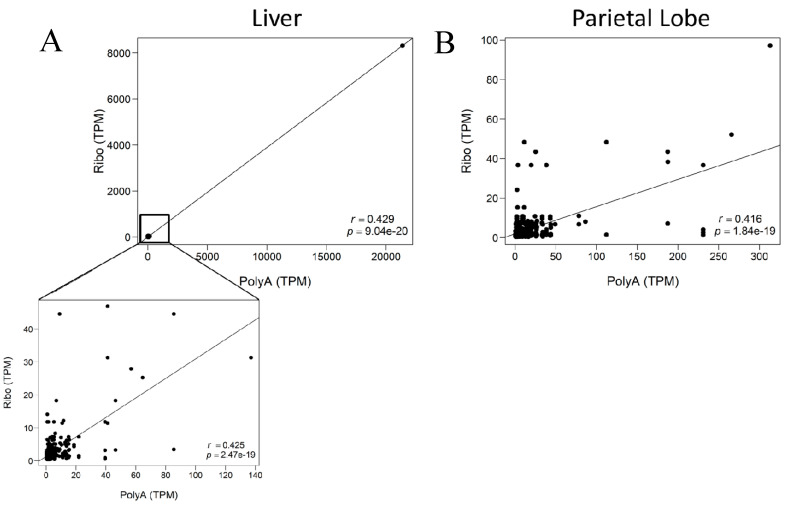
Correlation plots for lncRNA expression in AH1 with RefSeq annotated lncRNA. The x-axis indicates expression of the individual lncRNA in the poly-A^+^ selected library. The y-axis shows expression of the same lncRNAs in the rRNA-depleted library. (**A**) Liver (**B**) Parietal lobe of the cerebrum. Spearman correlation (r) and *p*-value in bottom right corner.

When evaluating the annotated lncRNA that had substantially higher TPMs in one library preparation as compared to the other, small nucleolar RNAs (snoRNAs) were consistently higher in rRNA-depleted libraries. There is minimal difference in the number of snoRNAs identified between each library preparation (Table S5); however there appears to be a large difference in the relative expression of snoRNAs in rRNA-depleted libraries. Limiting the correlation analysis to the EquCab3.0 RefSeq annotated lncRNA (https://ftp.ncbi.nlm.nih.gov/genomes/all/GCF/002/863/925/GCF_002863925.1_EquCab3.0/), which excludes snoRNAs, removed all transcripts that had high expression (>60 TPM) in the rRNA-depleted libraries and substantially disproportionate low expression in the poly-A^+^ selected libraries. However, the correlation between library preparations slightly decreased (Spearman r_(liver,subset)_ = 0.425, *p* = 2.47 × 10^−19^; Spearman r_(parietal)l_ = 0.416, *p* = 1.84^−19^; Figure 5 and Figure S3), but this is likely due to the decrease in number of transcripts used in the analysis.

## 3. Discussion

Long ncRNAs play a role in many cellular functions [1,2,3,4,5,6,7] and have been implicated in cancer [8,9,10] and neurodegeneration [11,12]. Additionally, lncRNAs may play a large role in athletic performance in the horse [22]. Thus, the goal of this study was to determine which RNA-seq library preparation method most reliably identifies lncRNA in horses. We hypothesized that rRNA-depleted libraries would be preferable as selection is not dependent on a poly-A tail. However, with an average coverage of 30 M reads, we identified more total lncRNA in the poly-A^+^ selected libraries, suggesting that poly-A^+^ selection may be more efficient at capturing lncRNAs than rRNA-depletion. It has been shown that some non-polyadenlyated lncRNAs are stabilized by other secondary structure features [23]. These features could have prevented the isolation of the associated transcripts during library preparation, leading to fewer lncRNAs being detected in the rRNA-depleted libraries. Additionally, the high TPM values obtained for snoRNAs suggest that these transcripts are overrepresented in rRNA-depleted libraries, preventing other ncRNAs from being identified. Previous studies on RNA-seq from human cell lines and blood have detected more or equal numbers of lncRNA in rRNA-depleted libraries [14,24,25,26], though one study explicitly reported that rRNA-depleted libraries yielded fewer usable reads than poly-A^+^ selection [14]. The study that found this included RNA from the colon, however this RNA sample was obtained directly from a commercial source and was not isolated by the researchers. Our study is unique in that all RNA was isolated by a single researcher from flash-frozen tissue collected from healthy, well-phenotyped individuals before proceeding to library preparation. This minimizes the potential variation from multiple individuals performing RNA isolations as well as any pathological variation. As a result, we did identify a strong correlation between biologic replicates.

While this study highlights many of the drawbacks of rRNA-depletion library preparation methods, poly-A^+^ selection has its own disadvantages. As seen here, some transcriptomic information is lost if it does not have a polyadenylated tail. Additionally, it is well known that poly-A^+^ selected libraries have a 3′-bias. In an effort to identify a library preparation method that avoids some of the biggest disadvantages of poly-A^+^ selection and rRNA-depletion, the rRNA-depletion protocol could be further optimized. Alternatively, a NuGEN Ovation v2 protocol, which utilized both random and oligo(dT) primers to remove rRNAs, performed well in lncRNA identification in one comparison study [27]. This method addressed the poly-A tail disadvantage; however, it performed poorly when looking at protein-coding transcripts and had a substantial 3′-bias [27]. In short, further research is needed to develop an improved RNA-seq library preparation protocol.

Investigating DE lncRNA between tissues for poly-A^+^ selection and rRNA-depletion as a proof of concept identified several lncRNA that are similar to lncRNA found in humans with similar expression differences between brain and liver tissues [20]. This suggests that both methods may be used to annotate lncRNA that is already known in other species. However, more lncRNA transcripts were unique to poly-A^+^ selected libraries than to rRNA-depleted libraries, indicating that, for a specific sequencing depth, poly-A^+^ selection may yield more informative lncRNA data.

When comparing poly-A^+^ selection and rRNA-depletion methods in humans, it is common to limit the comparison to already annotated lncRNA [14,24,25,26]. While this may be sufficient when using human data, there are not enough lncRNAs annotated in the equine reference genome to identify a substantial correlation between library preparation methods [14,24]. However, we can still observe a moderate correlation between library preparations. rRNA-depletion is often recommended for poor-quality RNA where a full transcript is likely not attached to a polyA-tail [28]. The RNA used in this study was of high quality and therefore our comparative results only apply to high-quality RNA library preparations.

As suggested in Scott et al. [13], library preparation does play a large role in the lncRNA that are detected. As such, rRNA-depleted datasets should not be considered equivalent to poly-A^+^ selected datasets. While this might not raise problems in the annotation of the equine genome, differential transcript expression studies between two equine populations would require additional biologic replicates to overcome the variation between library preparations. A primary advantage of using rRNA-depletion appears to be enhanced identification of snoRNAs. Data from human studies supports this. In a previous report using the HEK293 cell line, a snoRNA was one of the top three highly expressed lncRNA [25]. That particular study raised a valid concern that these highly expressed snoRNAs and similar transcripts lowered the sequencing depth for other RNAs [25]. Similarly, when using pooled blood RNA and a single colon RNA sample, a large portion of rRNA-depleted libraries consisted of a small number of lncRNAs and small RNAs (smRNAs) [14]. Therefore, for annotation of these RNAs, rRNA-depletion would likely be the most thorough. However, poly-A^+^ selection can identify these RNAs and may simply require deeper sequencing to better detect these transcripts. Previous study of HEK293 cells support this finding of highly expressed lncRNA from rRNA-depleted libraries also being present in poly-A^+^ selected libraries [25].

As only two tissues were used in this study, the results obtained here do not provide a thorough annotation of the equine genome. By evaluating eight different tissues, Scott et al. identified 20,800 putative lncRNA [13]. This number of putative lncRNA far exceeds what was identified in our study; however, tissues used in the Scott et al. study included both nervous and embryonic tissues, which likely have a substantially different lncRNA transcriptional profile as compared to adult horses [29]. In humans, rRNA-depletion has been reported to include more intergenic and intronic reads than poly-A^+^ selection [14]. Unfortunately, as many non-coding RNAs and untranslated regions are not identified in the horse, an accurate measure of the non-exonic reads in our dataset cannot be obtained. Similarly, we do not know the true distribution of lncRNA in horses. However, the identification of similar lncRNA that are differentially expressed between the liver and parietal lobe in both humans and horses suggest a potential method for future annotation.

Potential limitations of this study include the use of only two tissues from two biological replicates. Since the liver and parietal lobe are quite different in terms of cellular make-up complexity, they were considered to be good representative tissues. However, there could be some factors concerning RNA-seq library preparations we are not observing with the limited sample number. A limitation of the pipeline used is a combination of the strict filtering of predicted protein-coding sequences and an ineffective rescue of known lncRNA from human data. All transcripts with an open reading frame (ORF) were filtered out which likely excluded some lncRNA as there are reports of lncRNA with short ORFs in mice [30,31]. As noted from the increase in lncRNA after rescuing filtered out known lncRNA, a substantial number of lncRNA were incorrectly filtered out. An alternative pipeline may remove this part of the filter, though there is then the possibility of retaining unannotated or truncated protein-coding transcripts. Additionally, due to the low sequence conservation of lncRNA between horse and human [13], there are likely lncRNA that are not rescued as this step uses nucleotide BLAST (BLASTN). An improved rescue might utilize lncRNA known to be expressed in a specific tissue in a more thoroughly annotated species, such as human, and identify lncRNA in syntenic regions of the organism of interest, such as horse.

In summary, poly-A^+^ selection allowed for the identification of more lncRNA and missed fewer lncRNAs compared to rRNA-depletion. While changes to the pipeline could improve annotation, using poly-A^+^ selection in equine samples provides thorough identification of lncRNA.

## 4. Materials and Methods

### 4.1. Samples and Sequencing

Liver and parietal lobe of the cerebrum tissues from two healthy Thoroughbred mares was obtained from the functional annotation of the animal genome biobank [20] to investigate lncRNA expression in both a homogeneous and a complex tissue. RNA was isolated using a phenol-chloroform method with a column clean up. RNA quality was measured using an Agilent Bioanalyzer (RIN = 8.7). Two RNA-seq libraries were prepared from each tissue sample, one based on poly-A^+^ selection and the other using rRNA-depletion. Poly-A^+^ selected libraries were made using a strand-specific poly-A^+^ capture protocol (TruSeq Stranded mRNA, Illumina, San Diego, CA, USA). A bioanalyzer was used to ensure all poly-A^+^ selected libraries had adequate size distributions. The rRNA was depleted (Ribo-Zero, Illumina, San Diego, CA, USA) and prepared as strand-specific (TruSeq Stranded Total RNA Library pre kit, Illumina, San Diego, CA, USA). The libraries were size selected for 140 bp ± 10% fragments and sequenced on a HiSeq 4000 to an average depth of 30 M mapped reads (ERX2600970, ERX2600971). The reads are paired end and 125 bp long.

### 4.2. lncRNA Identification

The reads were trimmed with Sickle [32], mapped with STAR (2-pass) [33], map quality checked with samtools flagstat (>99% mapped and properly paired) [32], down-sampled to similar read counts across samples with samtools view [34], and annotated with Stringtie [35]. The EquCab3 reference genome and corresponding annotation was obtained from NCBI [36]. A lncRNA pipeline slightly modified from the one published by Scott and Mansour [13] was used to isolate the lncRNA and compare the two library preparation methods. Known protein coding transcripts were removed using a combination of filtering out any transcript GffCompare [37] identified as an exact match with protein coding transcripts from the reference and anti_join [38] against known protein coding transcript names. A histogram of TPMs was used to identify the cut-off to filter out the single exon transcripts with low TPMs that were categorized as false positives (TPM < 2) and to determine the TPM cut-off for the remaining transcripts (TPM < 0.4). As lncRNA are defined as longer than 200 bp, the transcripts were also filtered based on length. Remaining protein coding regions were computationally identified by combining predicted ORFs, protein domain models (Pfam) [39,40], and protein sequence database (hmmsearch; http://hmmer.org/). Additionally, the reads were BLAST’d (NCBI) against known human protein coding cDNA and protein peptide sequences. These findings were merged and filtered out. Known human lncRNA from the Ensembl ncRNA database (ftp.ensembl.org/pub/release-86/fasta/homo_sapiens/ncrna/Homo_sapiens.GRCh38.ncrna.fa.gz) were then compared to the filtered out coding regions and any matches were returned to the final lncRNA file. The lncRNA analysis and final bed files are detailed at https://github.com/ADahlgren/PolyA_ribozero.

### 4.3. Analysis

BEDtools intersect (version 2.29.2) [41] was used to identify lncRNA that do not overlap (by 50%) with the opposite library to identify the number lncRNA that were unique to each library preparation for each tissue. BEDtools intersect was also used to identify transcripts in one library that overlap with transcripts from another library (by 90%) and to identify transcripts that are likely RefSeq lncRNAs.

Correlation plots were made in Rstudio. The spearman rho (r) value and *p*-values were also calculated in Rstudio. EdgeR [42] was used to create MDS scaling plots based on log fold change to determine the role library preparation plays in the lncRNA transcriptome. It was also used to identify the most differentially expressed transcripts between liver and parietal lobe for each library preparation method, with *p* values corrected by a false discovery rate of <0.05. Principal component analysis was done using the dataset from EdgeR in Rstudio.

### 4.4. qRT-PCR

RNA from AH1 and AH2 was reverse transcribed into cDNA using SuperScript III (ThermoFisher; Waltham, MA, USA) according to the manufacturer’s instructions. Primers were designed using Primer 3 Plus (http://www.bioinformatics.nl/cgi-bin/primer3plus/primer3plus.cgi) to span two exons (Table S5). Endpoint PCR showed specific amplification of the correct product. qRT-PCR was performed on an AriaMx Real-time PCR System (Agilent; Santa Clara, CA, USA) using Brilliant III Ultra-Fast SYBR qPCR Master Mix (Agilent; Santa Clara, CA, USA). cDNA was pooled and serially diluted to ensure efficient amplification and the optimal dilution. All samples were run in triplicate and delta Cqs were calculated with *ACTB* as the reference gene.

## Supplementary Materials

The following are available online at https://www.mdpi.com/2311-553X/6/3/32/s1, Figure S1: Average number of protein-coding transcripts, Table S1: Number of lncRNA Used in Correlation Analysis by Tissue and Library Preparation, Table S2: lncRNA Removed in Biologic Replicate Correlation, Table S3: raw output from edgeR analysis, Figure S2: Bar graphs showing delta Cq in parietal lobe and liver for four lncRNA transcripts, Figure S3: Principle component plot of the lncRNA expression, Figure S4: Correlation plots for lncRNA expression in AH2 with annotated transcripts, Table S4: lncRNA annotated as snoRNA for each horse, tissue, and library preparation method, Figure S5: Correlation plots for lncRNA expression in AH2 with RefSeq annotated lncRNA, Table S5: qRT-PCR primer sequences.
