# Supplementary material for: Comparison of Poly-A+ Selection and rRNA Depletion in Detection of lncRNA in Two Equine Tissues Using RNA-seq
Source: Noncoding RNA. 2020 Aug 21;6(3):32. doi: 10.3390/ncrna6030032 (PMC7549351; doi:10.3390/ncrna6030032)
Supplement: Supplementary file 1 [file ncrna-06-00032-s001.zip › supp-re2.pdf]

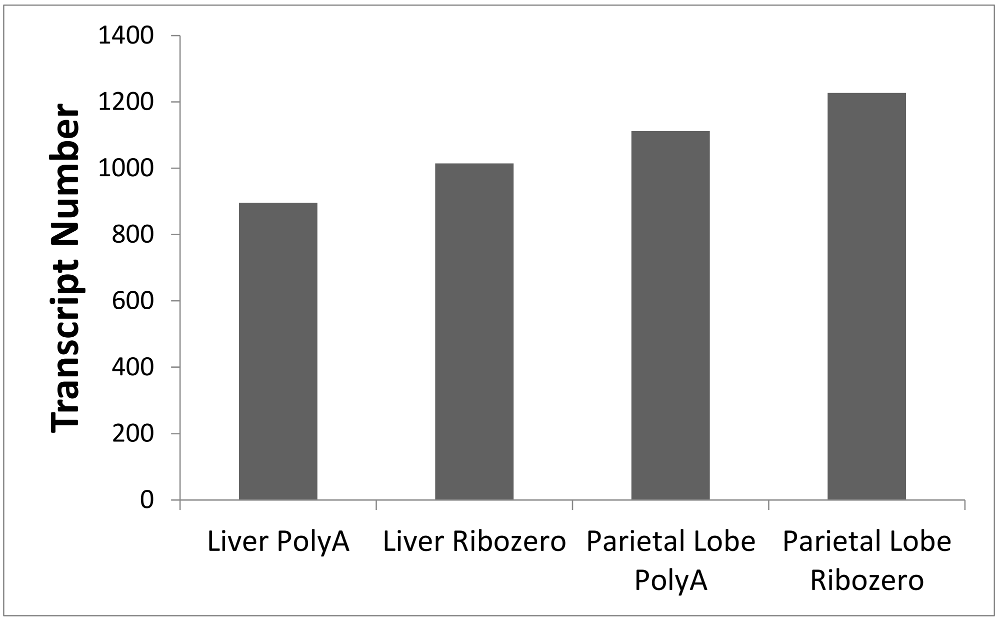

Figure S1. Average number of protein-coding transcripts. Bar graph showing the average number of protein-coding transcripts for each tissue and library preparation. Each bar is an average of the two horses.

Table S1: Number of lncRNA Used in Correlation Analysis by Tissue and Library Preparation

Tissue and Library Preparation	Number of lncRNA Used in Correlation
Liver PolyA	1934
Liver Ribozero	1208
Parietal Lobe PolyA	2371
Parietal Lobe Ribozero	1364

Table S2: lncRNA Removed in Biologic Replicate Correlation, Table S3: raw output from edgeR analysis

lncRNA Removed in Biologic Replicate Correlation			
Liver		Parietal Lobe	
PolyA	Ribozero	PolyA	Ribozero
chr7:25389286-25390378	chr31:8722887-8723106	chr1:112301900-112302558	chr1:21949607-21950018
chr27:34137241-34138781		chr10:76657675-76658212	chr1:112301900-112302558
		chr31:8722887-8723106	chr1:158746022-158746364
		chr31:16560679-16562097	chr27:5219606-5221622
			chr31:8722887-8723106

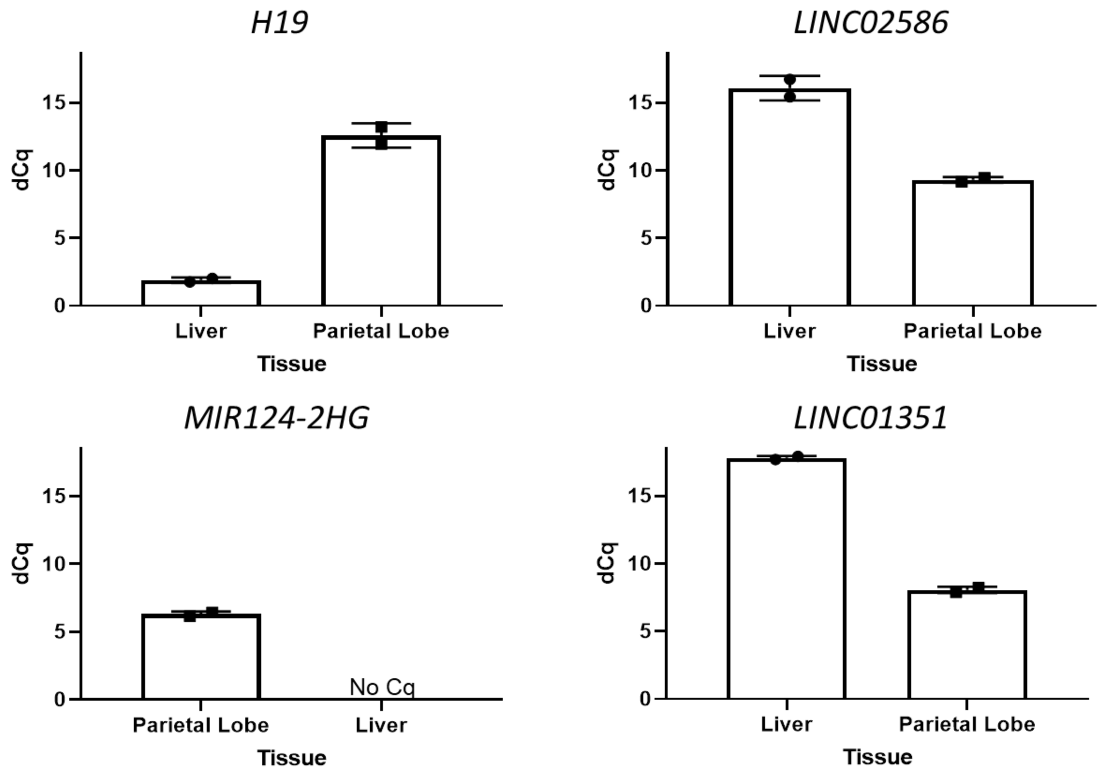

Figure S2: Bar graphs showing delta Cq in parietal lobe and liver for four lncRNA transcripts Bar graphs showing delta Cq in parietal lobe and liver for four lncRNA transcripts. Each data point represents one horse. *ACTB* was used as the reference gene.

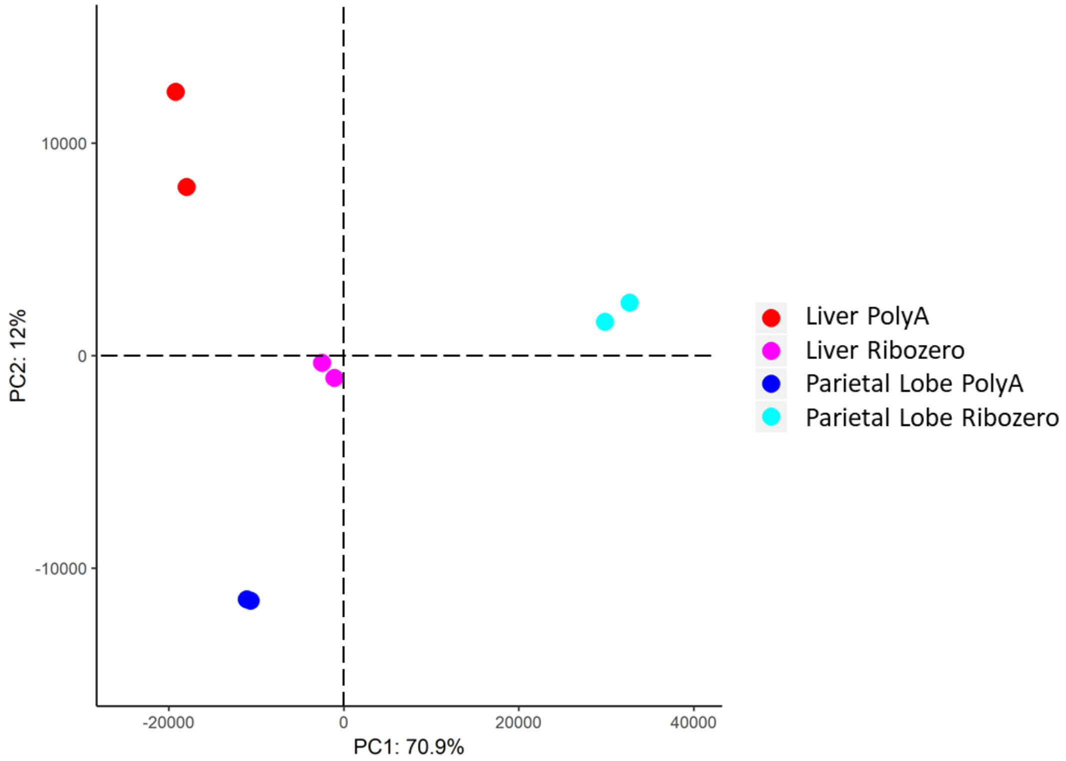

Figure S3: Principle component plot of the lncRNA expression. Principle component plot of the lncRNA expression from each tissue/library preparation for each horse

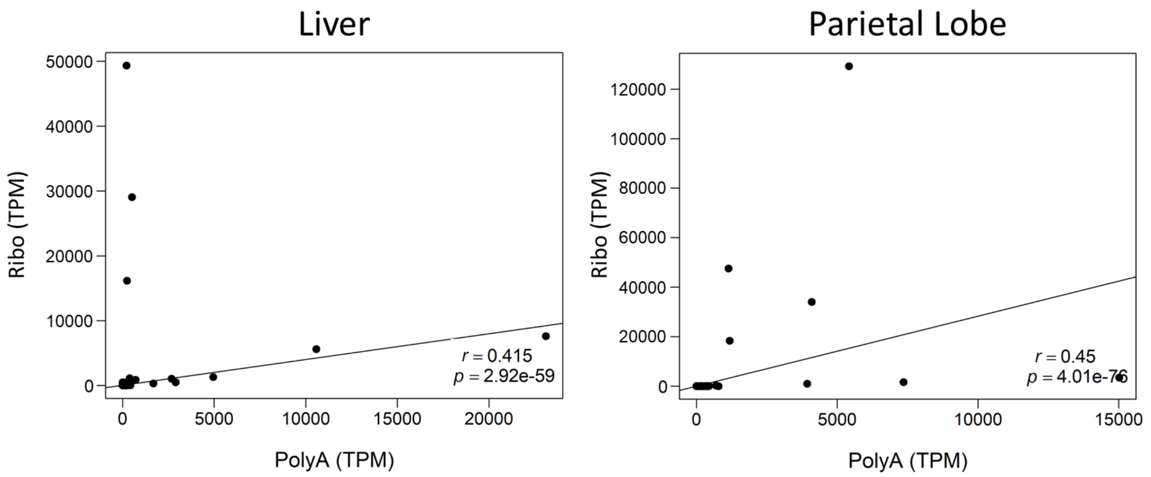

Figure S4: Correlation plots for lncRNA expression in AH2 with annotated transcripts, Correlation plots for lncRNA expression in AH2 with annotated transcripts. The x-axis indicates expression of the individual lncRNA in the poly-A^{+} selected library. The y-axis shows expression of the same lncRNAs in the rRNA-depleted library. A) Liver B) Parietal Lobe of the Cerebrum. Spearman correlation (r) and p-value in bottom right corner.

Table S4: lncRNA annotated as snoRNA for each horse, tissue, and library preparation method.

Horse	Tissue	Preparation method	lncRNA annotated as snoRNA
AH1	Liver	PolyA	178
		Ribozero	180
	Parietal Lobe	PolyA	201
		Ribozero	178
AH2	Liver	PolyA	213
		Ribozero	181
	Parietal Lobe	PolyA	237
		Ribozero	158

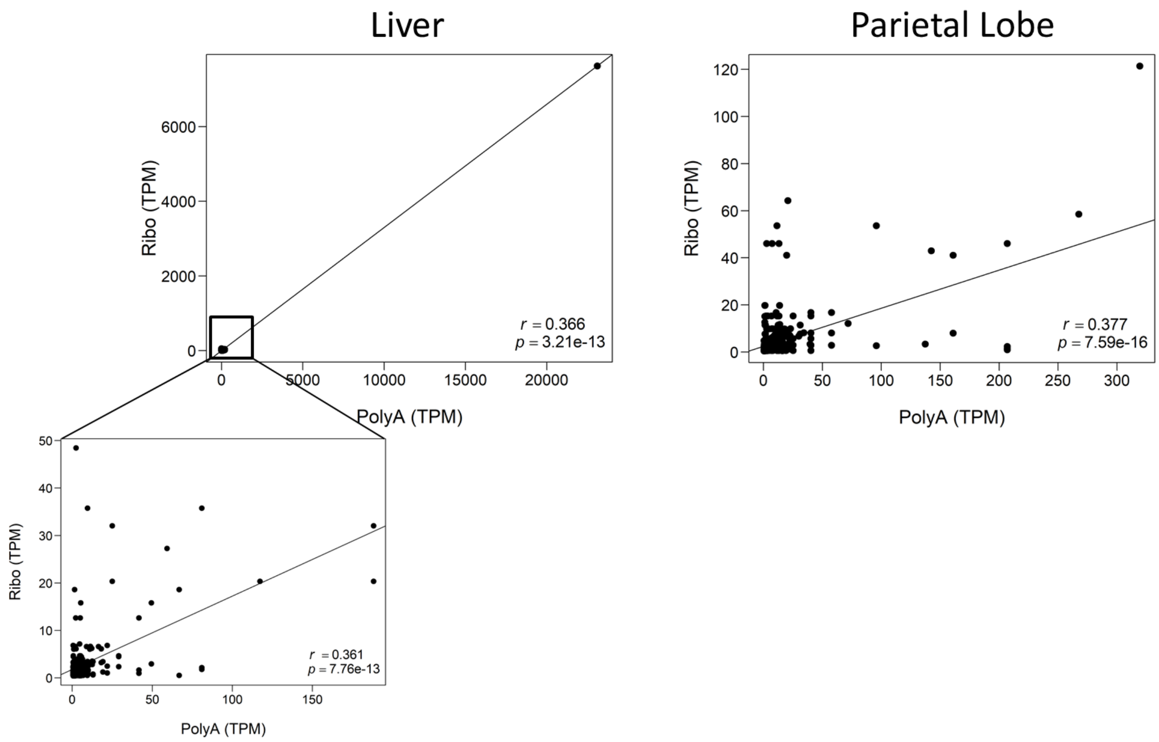

Figure S5: Correlation plots for lncRNA expression in AH2 with RefSeq annotated lncRNA. Correlation plots for lncRNA expression in AH2 with RefSeq annotated lncRNA. The x-axis indicates expression of the individual lncRNA in the poly-A^+ selected library. The y-axis shows expression of the same lncRNAs in the rRNA-depleted library. A) Liver B) Parietal Lobe of the Cerebrum. Spearman correlation (r) and p-value in bottom right corner.

Table S5: qRT-PCR primer sequences.

qRT-PCR Primer Sequences		
**Transcript**	**Forward Primer**	**Reverse Primer**
H19	GAAGAAGTCCGGGTTCCAA	CCCAAGAACCCTCAAGATGA
LINC02586	TCTGTCACAGTCACCGAAGC	ACCATCCAGATGGACAAGGA
MIR124-2HG	AGAGCCGGTCTAGCTCAGAGA	GAGACGCGGATTACCCAAAC
LINC01351	TATCCCGATACCGCACAAAC	TTCCACTCTACCTTTCCCTCCT
ACTB	AAGGAGAAGCTCTGCTATGTCG	GGGCAGCTCGTAGCTCTTC
